# Determination of Iodine Concentration in Commonly Consumed Salt and Its Potential Impact on Household Consumers: An Examination and Assessment of Consumed Salt

**DOI:** 10.1002/puh2.70012

**Published:** 2024-12-02

**Authors:** Md Shahedul Islam, Mohammad Asadul Habib, Mohammad Anwar Ul Alam, Oumma Halima, Nusrat Parvin, Md. Rezaul Karim, Abdur Rahman Shanna, Rifat Jahan Romel, Abdur Rahman Sakib, Mohammad Ariful Islam

**Affiliations:** ^1^ Department of Food Technology and Nutrition Science Noakhali Science and Technology University Noakhali Bangladesh; ^2^ Department of Food Science Cornell University New York New York USA; ^3^ Institute of Nutrition and Food Science University of Dhaka Dhaka Bangladesh; ^4^ Department of Biochemistry and Molecular Biology Noakhali Science and Technology University Noakhali Bangladesh

**Keywords:** branded salts, goiter, iodine deficiency disorder (IDD), iodized salt, iodometric titration, raw salt, salt consumption

## Abstract

**Introduction:**

Iodine deficiency disorder (IDD) can lead to health issues as it is necessary for metabolic functions. This study investigated the iodine content of commonly consumed salt and the potential impact on daily iodine requirements and IDDs.

**Methods:**

A cross‐sectional survey was employed using multistage cluster sampling among 240 households, dividing them into eight clusters from four stratified areas. The sampled salts were analyzed using the World Health Organization's iodometric titration method.

**Results:**

The study revealed that most salt samples from the studied brands had an iodine content within the Bangladesh Standards and Testing Institution (BSTI) recommended range of 20–50 ppm, with two exceptions. The study found that most socioeconomic groups in cities, towns, and semirural areas meet their daily iodine requirements at over 100%. At the same time, many rural homes, regardless of income, fail to meet these requirements. However, the study found iodized salt in 97.9% of all surveyed households. Although 48.3% of unions, that is, rural respondents, was knowledgeable about the existence of iodized salt, 66.7% of respondents was not aware of the health benefits of iodized salt. Surprisingly, 63.3% of the town's household members and 61.7% of the rural population still consume raw salt. Additionally, 79.6% of individuals reported having no IDDs. However, only 44.2% of thyroid patients used iodine supplements.

**Conclusion:**

Although salt usage and consumption practices are satisfactory, there are still a few concerns about the 100% iodization of branded and raw salts. As a result, many households need help to satisfy their daily iodine requirements.

## Graphical abstract

•Most salt samples met the recommended iodine levels, yet a significant number of rural households failed to meet daily iodine requirements. Although iodized salt was prevalent in 97.9% of surveyed households, awareness of its health benefits was low, especially in rural areas.

•Notably, a large percentage of town and rural households still consumed raw salt. Despite satisfactory salt usage, the study highlighted concerns about complete iodization and its effect on preventing iodine deficiency disorders.

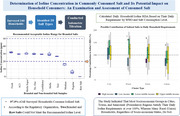

## 1 Introduction

Iodine deficiency poses a significant global public health concern [1] due to its impact on thyroid hormone production, which is crucial for normal brain development [2]. Iodine deficiency disorder (IDD) challenges bioavailability or insufficiency and affects 13% of the global population across 130 nations [3, 4]. The National Institutes of Health (NIH) classifies goiter [5] as the preeminent symptom of IDD. IDD [6], a congenital disorder that can result in dwarfism, mental disorders, spastic weakness, paralysis, goiter, stillbirths, and miscarriages, as well as thyroid deficiencies in neonates and juveniles, has had a significant impact on Bangladesh.

Over the last three decades, there has been significant progress in the global effort to combat various preventable diseases. The prevalence of iodine‐deficient nations decreased from 54 to 32 between 2003 and 2011, whereas the number of countries with sufficient iodine intake increased from 67 to 105 in 2011. On the basis of the latest data in 2011, 71% of people globally had access to iodized salt [7]. Although there is no country‐specific data available in the literature regarding the recent IDD prevalence in Bangladesh, a recent study revealed that a considerable proportion of adolescent girls in peri‐urban areas were diagnosed with iron deficiency anemia, with a prevalence rate of 32% [8].

The World Health Organization (WHO) pressures the necessity for iodized salt, which may provide iodine levels between 15 and 40 parts per million (ppm) and be available in households [3]. The iodization of all salt intended for human consumption became mandatory in 1989 when Bangladesh implemented the IDD Prevention Act [9]. Besides, iodized salt is still a mainstay for supplying the daily iodine requirements [10].

However, on the basis of the recent data shared by UNICEF Bangladesh in 2012, only 57.6% of Bangladeshi households use sufficient iodized salt, indicating a crucial limitation of up‐to‐date research on its availability and usage. That may leave 68 million people susceptible to diseases caused by inadequate iodine consumption [9]. The Bangladesh National Salt Iodization Survey (NSIS) 2015 reported that only 65% of households in Bangladesh have access to iodized salt, whereas another recent research found that just 50.5% of households had enough access to iodized salt [11].

This research study focuses on household consumption patterns of iodized salt and respondents’ knowledge regarding its usage and IDDs.

## 2 Methods and Materials

### 2.1 Study Design and Sampling

The cross‐sectional research included 240 families from 8 clusters of 4 strata (city, town, pourashava, and union) between October and November 2023. The researchers selected study participants at random using a thorough multistage cluster sampling procedure. Starting from the center point of each cluster, they skipped every second family and chose the next one.

### 2.2 Study Site and Sample Size Selection

The research area included eight clusters located in four different places within the Chattogram administrative division: Chattogram and Cumilla (cities), Lakshmipur and Feni (towns), Maijdee and Chakaria (pourashavas), and Dharmapur and Ewazbalia (unions) (Figure S1). The popular formula for proportions was utilized to calculate the sample size on the basis of the latest iodized salt consumption rate [12].

This study uses a proportion rate of 80.3%, the 2011–2012 iodized salt consumption rate [13]. Besides, the precision variable “*d*” was set to 0.05. As a result, the formula yields a sample size of 244. However, we selected 240 households to guarantee equal distribution across eight clusters. The study followed UNICEF's Monitoring of Salt Iodization Program and Population Iodine Status Standards [14].

### 2.3 Questionnaire Development

A semi‐structured questionnaire was employed to collect data from clustered households—the first section deals with sociodemographic variables, and the second looks at salt consumption. The questionnaire concluded with Knowledge, Attitude, and Practices (KAP) regarding IDD. The demographic section was adapted from the Bangladesh Bureau of Statistics (BBS) Population and Housing Census 2022 [15] and the Bangladesh Demographic and Health Survey 2022 [16]. Haque et al. [17] defined as 8500 or less BDT per month, lower middle income as 8501–32,000, upper middle income as 32,001–100,000, and high income as 100,000 or more. Karmakar et al. [18] guided the household salt consumption section. The KAP section used UN FAO guidelines [19] and Goris et al.’s Papua New Guinea study [20]. Three independent experts in cross‐sectional research have validated the prepared questionnaire. Before beginning the field survey, a pilot survey was conducted beyond selected areas, covering 10% [21] of the sample size.

### 2.4 Data Collection Procedure

The field interviewers obtained informed consent from all study participants with a signed form. Few female responses were received, so we interviewed the person who made daily meals. Senior male members conducted the interviews when no females were present. Following a few earlier studies, we meticulously recorded the survey data using the KOBO Toolbox [21, 22, 23]. All data have been transformed into suitable codes, and no personal identification has been recorded to ensure confidentiality.

### 2.5 Household RDA Calculation of Iodine

The WHO advises a daily dose of 130 mcg of iodine for infants aged 7–12 months, 90 mcg for children aged 1–8 years, 150 mcg for adolescents aged 14–18 and adults, 220 mcg for pregnant women, and 290 mcg for lactating women [5]. Using the following equation, we generated each household member's Recommended Dietary Allowance (RDA) based on the WHO's recommendations:

$$\begin{array}{ccc} & & \mathrm{Daily}\;\mathrm{Household}\;\mathrm{Iodine}\;\mathrm{Requirement}\\ & & \;=\mathrm{Individual}\;\mathrm{Iodine}\;\mathrm{Requirement}\;\mathrm{of}\;\mathrm{Household}\;\mathrm{Member}1\\ & & \;+\;\mathrm{Member}\;2+\;\mathrm{Member}\;3+\cdots \end{array}$$

We contrasted the daily iodine intake with the total family requirement. The percentage results made it possible to evaluate and compare the household's iodine consumption to WHO recommendations.

### 2.6 Collection of Salt Samples

The researchers selected 10 salt samples via survey according to consumption trends in selected clusters. Nine of the samples had branding, whereas one did not. These samples, intended to represent typical home salt types, came from the local market.

### 2.7 Analysis of Salt Samples

After collection, researchers carefully unpacked salt samples and placed them in vacuum containers with matching specifications. For anonymity and impartiality, labels concealed their allocation during analysis. These sampling salts were tested for iodine using WHO standards in “Assessment of IDDs and Monitoring Their Elimination” [3, 24] (see Supporting Information).

### 2.8 Statistical Analyses

Ten percent of the random sample has been checked carefully. Cross‐tabulation and Pearson chi‐square testing were used in SPSS (version 26) to examine the association among sociodemographic variables with cluster types. Iodine concentration in mg per 100 g, also in parts per million (ppm), and the percentage contribution to daily household iodine requirements were analyzed and visualized with R Studio (version 4.3.2). To run the linear regression sociodemographic factors, that is, cluster type (1 for “union,” 2 for “pourashava,” 3 for “town,” and 4 for “city”), education (1 for “no education” to 4 for “higher education” respectively), occupation (l–9 for others, retired, farmer, day labor, business, nongovernment employee, government employee, student, and housewives, respectively), socioeconomic status (1 for “low income” to 4 for “high income,” respectively), religion (1 for “Buddhism,” 2 for “Hinduism,” and 3 for “Islam”), family type (1 for “single” and 2 for “joint family”), knowledge score, and practices score (based on hedonic score) that affected the percentage contribution to RDA iodine intake have been coded accordingly.

## 3 Results

### 3.1 Socioeconomic and Demographic Information of the Study Population

The study involved 240 participants from various locations, mostly female. The majority completed secondary school in cities and towns, with pourashava having the highest rate. Most respondents were homemakers, and Islam was the most predominant religion. The majority of respondents in cities and towns (urban clusters) were upper middle class, whereas in pourashava (semi‐urban clusters) and union (rural clusters), they were lower middle class. All clusters had a high prevalence of single families, primarily in cities. Specifically, all households surveyed from the city clusters were single (see Table S1).

### 3.2 Iodine Concentration Analysis

**FIGURE 1:**
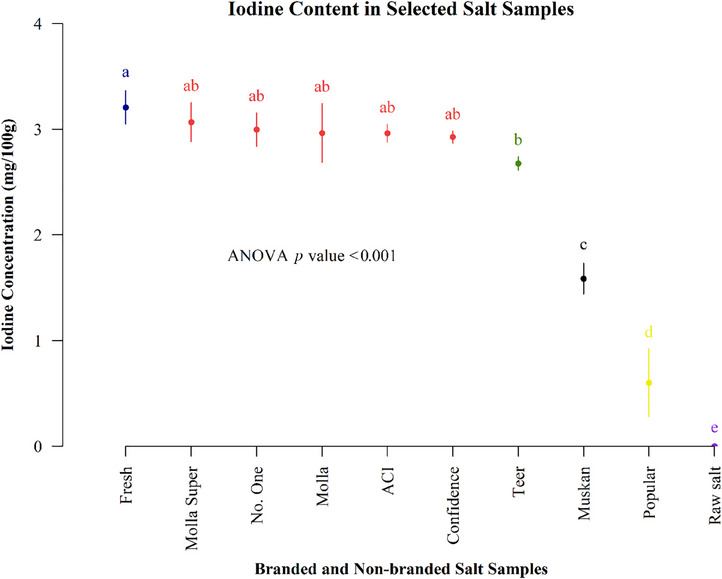
Iodine content in selected branded and non‐branded salts.

Figure 1 lists that the content of popular brand salt was the lowest, and that of fresh brand salt was the greatest. The raw sample's iodine content was nonexistent. The Teer, ACI, Molla Super, No. One, Molla, and Confidence brands have the second‐highest iodine levels. The Fresh brand shares similarities with ACI, Molla Super, No. One, Molla, and Confidence, but there are significant differences from other brands (*p* < 0.01).

**FIGURE 2:**
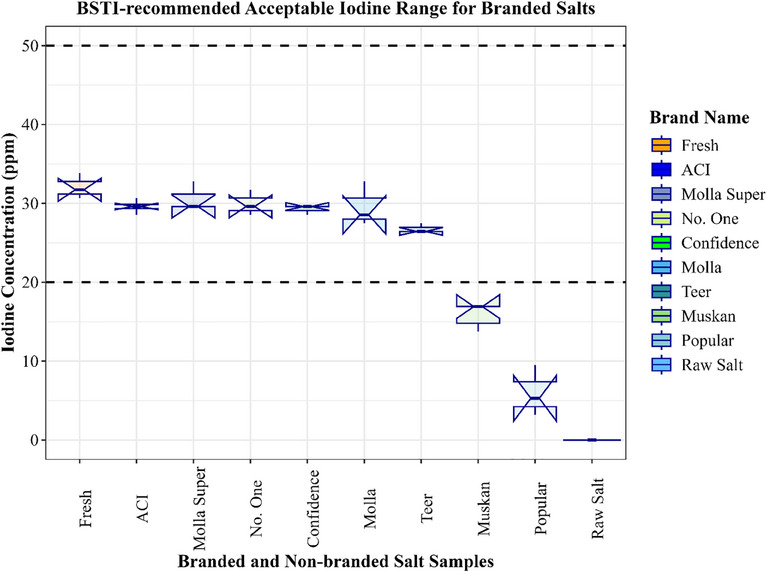
Mean concentration of sampled salts (in ppm). BSTI, Bangladesh Standards and Testing Institution.

The Bangladesh Standards and Testing Institution (BSTI) [25] has established a suggested range of 20–50 parts per million (ppm) for iodine concentrations in salt samples. Fresh branded salt has the highest iodine concentration, measuring 32.1 ppm. Muskan and Popular brands have iodine levels that fall significantly below the 20‐ppm BSTI threshold (Figure 2).

### 3.3 Percent Consumption of Iodine to Daily RDA by the Households

**FIGURE 3:**
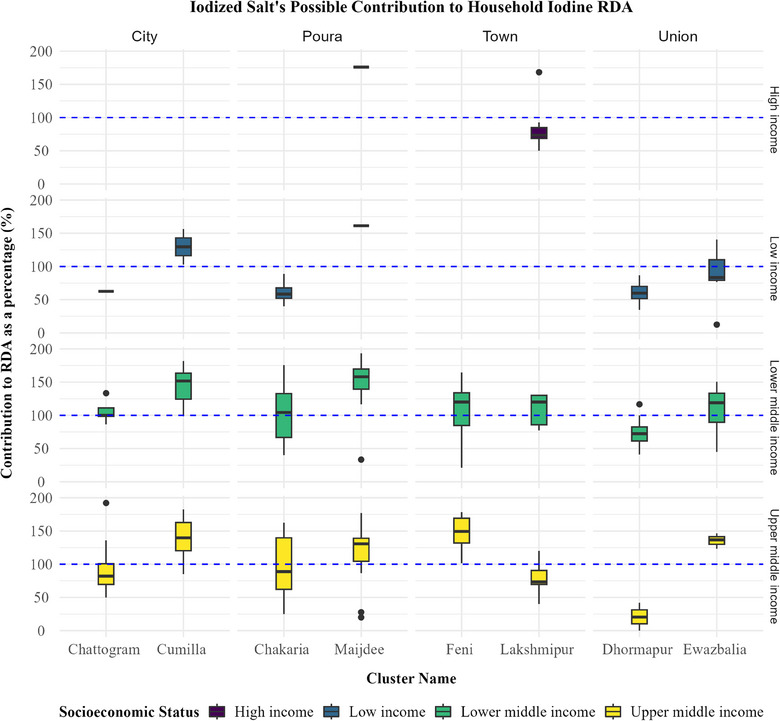
Estimated percent contribution to the daily household RDA of iodine.

Lower and higher middle‐income families may achieve their daily iodine needs at levels over 100% via salt consumption. Most low‐income urban and rural populations need help meeting their RDA. Low‐income households in one city (Cumilla) and one union (Ewazbalia) can satisfy their RDA. Lower‐ and upper‐middle‐income urban and semi‐urban families can meet their daily iodine requirements. Households in rural unions of all socioeconomic levels struggle to satisfy their daily iodine requirements from iodized salt (Figure 3).

### 3.4 Respondent's Knowledge About Iodized Salt and Iodine Deficiency Diseases

The study found that most pourashava, town, and city residents cook essential food items with iodized salt. The existence of iodized salt and its health benefits were unknown to about and more than half of union cluster respondents, respectively. The majority of urban (city and town), suburban (pourashava), and rural (union) households buy packaged salt for cooking; the proportion is 100% among the households from towns. Most households in each cluster store salt with a lid, except 15% in pourashava and 16.7% in union. Raw salt consumption habit is comparatively lower in city and pourashava clusters, whereas the habit is higher in town and union clusters. Between 5% and 10% of pourashava and city respondents had a thyroid disease history. Respondents from both clusters take iodine supplements, mostly on prescription. However, 18.3% of rural union respondents needed help getting iodized salt (Table 1).

**TABLE 1:** Respondent's knowledge about iodized salt and iodine deficiency diseases among the population of different clusters.

KAP questions	City (*N* = 60), *n* (%)	Town (*N* = 60), *n* (%)	Pourashava (*N* = 60), *n* (%)	Union (*N* = 60), *n* (%)	*p*
Knowledge about existence of iodized salt					
Yes	57 (95)	51 (85)	53 (88.3)	29 (48.3)	0.000*
No	3 (5)	9 (15)	7 (11.7)	31 (51.7)	
Knowledge about health benefits of iodized salt					
All the above	38 (63.3)	36 (60)	14 (23.3)	1 (1.7)	0.000*
Don't know	4 (6.7)	9 (15)	12 (20)	40 (66.7)	
Importance for mental development	1 (1.7)	0 (0)	0 (0)	0 (0)	
Prevents goiter	17 (28.3)	15 (25)	30 (50)	18 (30)	
Others	0 (0)	0 (0)	4 (6.7)	1 (1.7)	
Habit of using salt to cook the main meal					
Yes	60 (100)	59 (98.3)	60 (100)	57 (95)	0.000*
No	0 (0)	1 (1.7)	0 (0)	3 (5)	
Type of packaging usually used to buy salt					
Do not buy salt	0 (0)	0 (0)	1 (1.7)	0 (0)	0.000*
No answer	1 (1.7)	0 (0)	0 (0)	0 (0)	
Loose salt	0 (0)	0 (0)	1 (1.7)	0 (0)	
Poly bag with labeling	59 (98.3)	60 (100)	44 (73.3)	60 (100)	
Poly bag without labeling	0 (0)	0 (0)	14 (23.3)	0 (0)	
Salt preservation type					
Closed container with lid	59 (98.3)	60 (100)	51 (85)	50 (83.3)	0.000*
Open container without lid	1 (1.7)	0 (0)	9 (15)	10 (16.7)	
Salt type usually used by household					
Don't know	1 (1.7)	0 (0)	2 (3.3)	1 (1.7)	0.000*
Iodized	59 (98.3)	60 (100)	57 (95)	59 (98.3)	
Not iodized	0 (0)	0 (0)	1 (1.7)	0 (0)	
Habit of consuming raw salt during eating					
No	31 (51.7)	19 (31.7)	18 (30)	6 (10)	0.000*
Yes	23 (38.3)	38 (63.3)	27 (45)	37 (61.7)	
Sometimes	6 (10)	3 (5)	15 (25)	17 (28.3)	
Family history of thyroid disorder					
Don't know	11 (18.3)	8 (13.3)	3 (5)	18 (30)	0.000*
No	43 (71.7)	52 (86.7)	54 (90)	42 (70)	
Yes	6 (10)	0 (0)	3 (5)	0 (0)	
History of taking iodine supplement in the previous 3 months					
Don't know	29 (48.3)	37 (61.7)	13 (21.7)	30 (50)	0.000*
Never took	27 (45)	23 (38.3)	26 (43.3)	30 (50)	
Taking now	4 (6.7)	0 (0)	6 (10)	0 (0)	
Took before	0 (0)	0 (0)	15 (25)	0 (0)	
If took, who prescribed the supplement?					
Did not take	56 (93.3)	60 (100)	55 (91.6)	60 (100)	0.043*
Doctor/Medicine specialist	3 (5)	0 (0)	1 (1.7)	0 (0)	
Endocrinologist	1 (1.7)	0 (0)	1 (1.7)	0 (0)	
Own initiative	0 (0)	0 (0)	3 (5)	0 (0)	
Difficulties to buy and use iodized salt					
Difficult	0 (0)	0 (0)	0 (0)	1 (1.7)	0.000*
Don't know/No answer	9 (15)	2 (3.3)	1 (1.7)	2 (3.3)	
Not so difficult	51 (85)	58 (96.7)	56 (93.3)	46 (76.7)	
So‐so difficult	0 (0)	0 (0)	3 (5)	11 (18.3)	

*Note:* In this case, asterisks (*) highlight that there is a significant association between various KAP indicators and cluster types (Pearson chi‐square *p* value < 0.05).

### 3.5 Factors Affecting the RDA of Iodine at the Household Level

Household iodine intake differed greatly by practice score, family type, and education. Households with higher education and practice ratings contributed less iodine to their RDA. Higher educational levels correlate with a decline in iodine intake via salt consumption, leading higher educated individuals to seek alternative sources. The link between salt iodine consumption and socioeconomic status is also decreasing, but it is not statistically significant. Iodine intake is higher in nuclear households than in joint families (Table 2).

**TABLE 2:** Multiple linear regressions to identify demographics and socioeconomic variables that affect the per cent contribution to the daily RDA of iodine.

Demographics and socioeconomic variables	Percent contribution to the daily RDA of iodine			
	*B*	SE	*T*	*p*
Cluster type	4.0	3.2	1.2	0.209
Education	7.9	3.5	2.3	0.025*
Occupation	−0.5	1.6	−0.3	0.768
Socioeconomic status	−4.3	4.6	−0.9	0.353
Religion	0.0	10.5	0.0	1.000
Family type	−23.1	9.4	−2.5	0.015*
Knowledge score	0.7	1.9	0.4	0.726
Practices score	−5.5	2.7	−2.1	0.040*

*Note:* Here, the standardized coefficient is denoted by *B*. “SE” stands for the regression's standard error. The coefficient divided by its standard error is the “*T*” statistic.

Asterisks (*) highlight the statically significant value.

## 4 Discussion

This study revealed that most branded salts in Bangladesh satisfy the recommended iodine level set by regulatory bodies, except two. Although variations are based on residential and socioeconomic status (education, family type, and household practice), almost half of the studied households can meet daily iodine needs. However, all KAP indicators vary among cluster types.

Estimates suggested that approximately 2.5 billion individuals worldwide experienced insufficient iodine intake, with Southeast Asia, including Bangladesh, being particularly affected, with about 313 million people affected [1]. However, recent estimations by UNICEF indicate a positive shift, leaving approximately 1 billion individuals consuming non‐iodized salt. The present study estimated that, on average, 97.9% of surveyed households used iodized salt in their food preparation. Similar findings have been found in East Asia, the Pacific, and South Asia, showing extensive implementation of iodized salt, with 92% and 90% coverage rates, respectively [26].

Concerns arise regarding the recommended iodine level in branded salt products available in the market and consumers’ ability to meet their daily iodine requirements through salt consumption. Some research conducted in Bangladesh has raised concerns about certain well‐known salt brands lacking sufficient iodine content as stipulated by regulatory agencies [25, 27, 28]. The WHO recommends iodine fortification of food‐grade salt for household and food processing purposes to prevent and manage IDD [29]. According to WHO, UNICEF, and ICCIDD [30], branded salts should have an iodine content of 20–40 ppm during production, whereas the BSTI sets the acceptable range to be between 20 and 50 ppm [25]. However, this study revealed that two samples failed to meet the prescribed threshold of more than 20 ppm. It is not easy to assume the exact causes behind this, but temperature and light were noted to impact salt iodine quality. Even though these issues were raised, the study showed that the approximate daily contribution to meeting iodine requirements was positive. Three study locations met daily consumption needs, whereas households in the union (rural) clusters fell below 100% of RDA. Along with some other studies [25, 28], this study found that educational level, employment status, household practices, and family type significantly affect household iodine intake.

The KAP findings illuminate participants’ knowledge, attitudes, and behaviors regarding iodized salt and IDD. Overall, iodized salt was familiar to 83.3% of the study participants, and 72.9% was aware about its health benefits. An Indian study found 68.9% familiarity with iodized salt and 58.9% awareness of its benefits [18]. Most households (98.3%) used salt in cooking; 92% bought it in labeled polybags, and 91.7% stored it in lidded containers. At the same time, another study showed that about 75% of respondents needed to be made aware of the storage of iodized salt [31]. Only 46.5% of households in India consumed iodized salt, whereas this study found that the value is 97.92% [32]. Only a few (0.4%) studied households had trouble getting and using iodized salt; two other studies also found that some respondents face problems with purchasing iodized salt [32, 33].

Overall, this study may send a significant message to policymakers to rethink and reshape salt iodization regulation, as a few renowned salt brands could not maintain the usual standards. Besides, this may also aid in conveying a clear message about household‐level iodine consumption scenarios and current IDD prevalence. However, due to the small sample size in a particular administrative area and the limited number of salt samples analyzed, it is tough to estimate a general assumption for the scenario of the whole country. Thus, we recommend conducting further studies considering the limitations, focusing on preservation and cooking loss, and measuring urinary iodine concentration (UIC) in a real‐life scenario among household‐level consumers.

## 5 Conclusion

According to the study, a few well‐known salt brands in Bangladesh did not contain the recommended amount of iodine. Rural residents may need help getting enough iodine in their diets on a daily basis. We should enact laws prohibiting the sale of salt without iodization and ensure that all branded salts contain the recommended iodine concentration. More research on individual‐level iodine use, particularly urinary iodine levels, is necessary to determine the effectiveness of iodine interventions and develop targeted public health programs.

## Author Contributions

**Md. Shahedul Islam**: conceptualization, methodology, data curation, investigation, writing–original draft. **Mohammad Asadul Habib**: writing–review and editing, methodology, formal analysis, supervision, investigation. **Mohammad Anwar Ul Alam**: methodology, writing–review and editing, supervision, formal analysis. **Oumma Halima**: methodology, formal analysis, supervision, writing–review and editing. **Nusrat Parvin**: data curation, validation, writing–original draft. **Md. Rezaul Karim**: methodology, formal analysis, data curation, visualization. **Abdur Rahman Shanna**: methodology, validation, visualization, data curation. **Rifat Jahan Romel**: methodology, data curation, formal analysis, visualization. **Abdur Rahman Sakib**: methodology, data curation, validation, visualization. **Mohammad Ariful Islam**: methodology, data curation, validation, visualization.

## Ethics Statement

This study involved laboratory analysis of salt samples and a salt consumption scenario based on household‐level consumption. Prior to collecting field data from study areas, researchers obtained necessary ethical approval from the Noakhali Science and Technology University Ethical Committee (NSTUEC) under grant number NSTU/SCI/EC/2023/185.

## Conflicts of Interest

The authors declare no conflicts of interest.

## Supporting information



Supporting Information

## Data Availability

Data will be made available upon reasonable request.
