# Supplementary material for: Determination of Iodine Concentration in Commonly Consumed Salt and Its Potential Impact on Household Consumers: An Examination and Assessment of Consumed Salt
Source: Public Health Chall. 2024 Dec 2;3(4):e70012. doi: 10.1002/puh2.70012 (PMC12039590; doi:10.1002/puh2.70012)
Supplement: Supplementary file 1 — Supporting Information [file PUH2-3-e70012-s001.docx]

**Supplementary Table-S1:** Socio-economic and demographic information of the study population

| Characteristic | | City (N= 60), n (%) ^b^ | Town (N= 60), n (%) ^b^ | Pourashava (N= 60), n (%) ^b^ | | Union (N= 60), n (%) ^b^ |
| --- | --- | --- | --- | --- | --- | --- |
| Respondents Age (Mean± SD) | | 36 ± 8.3 | 38.1 ± 10.8 | 30.9 ± 12.5 | | 35.1 ± 11.8 |
| Gender ^a^ | Male | 3 (5) | 1 (1.7) | 6 (10) | | 13 (21.7) |
|  | Female | 57 (95) | 59 (98.3) | 54 (90) | | 47 (78.3) |
| Education ^a^ | No education | 1 (1.7) | 1 (1.7) | 2 (3.3) | | 14 (23.4) |
|  | Primary | 2 (3.3) | 8 (13.3) | 4 (6.7) | | 33 (55) |
|  | Secondary | 17 (28.3) | 19 (31.7) | 39 (65) | | 11 (18.3) |
|  | Higher Secondary | 25 (41.7) | 20 (33.3) | 7 (11.7) | | 2 (3.3) |
|  | Higher Education | 15 (25) | 12 (20) | 8 (13.3) | | 0 (0) |
| Occupation of respondent ^a^ | Business | 2 (3.3) | 4 (6.7) | 1 (1.7) | | 3 (5) |
|  | Day Labor | 0 (0) | 0 (0) | 1 (1.7) | | 3 (5) |
|  | Farmer | 0 (0) | 0 (0) | 0 (0) | | 7 (11.6) |
|  | Government Employee | 3 (5) | 15 (25) | 1 (1.7) | | 0 (0) |
|  | Housewife | 49 (81.7) | 40 (66.6) | 19 (31.6) | | 43 (71.6) |
|  | Non- Government Employee | 1 (1.7) | 0 (0) | 5 (8.3) | | 1 (1.7) |
|  | Old Age/ Retried | 0 (0) | 0 (0) | 0 (0) | | 1 (1.7) |
|  | Students | 5 (8.3) | 0 (0) | 33 (55) | | 1 (1.7) |
|  | Others | 0 (0) | 1 (1.7) | 0 (0) | | 1 (1.7) |
| Religion ^b^ | Islam | 54 (90) | 56 (93.3) | 58 (96.6) | | 59 (98.3) |
|  | Hinduism | 6 (10) | 4 (6.7) | 2 (3.3) | | 0 (0) |
|  | Buddhism | 0 (0) | 0 (0) | 0 (0) | | 1 (1.7) |
| Socio- Economic status ^a^ | Low Income | 3 (3.3) | 0 (0) | 5 (8.3) | | 13 (21.7) |
|  | Lower Middle Income | 15 (25) | 21 (35) | 36 (60) | | 41 (68.3) |
|  | Upper Middle Income | 42 (70) | 32 (53.3) | 18 (30) | | 6 (10) |
|  | High income | 0 (0) | 7 (11.7) | 1 (1.7) | | 0 (0) |
| Marital Status ^a^ | Married | 54 (90) | 60 (100) | 26 (43.3) | | 58 (96.6) |
|  | Single | 5 (8.3) | 0 (0) | 33 (55) | | 1 (1.7) |
|  | Widow/ Divorce | 1 (1.7) | 0 (0) | 0 (0) | | 1 (1.7) |
| Number of Household member ^a^  (Mean± SD) | | 4.2 ± 1.0 | 3.8 ± 1.3 | | 4.7 ± 1.6 | 4.2 ± 1.3 |
| Family Status ^a^ | Joint Family | 0 (0) | 5 (8.3) | 8 (13.3) | | 8 (13.3) |
|  | Single Family | 60 (100) | 55 (91.7) | 52 (86.7) | | 52 (86.7) |

Here, values with distinct superscripts (a, b) in rows and columns indicate a statistically significant relationship between demographic variables and cluster types (P-value<0.05).

**Study are map:**

**
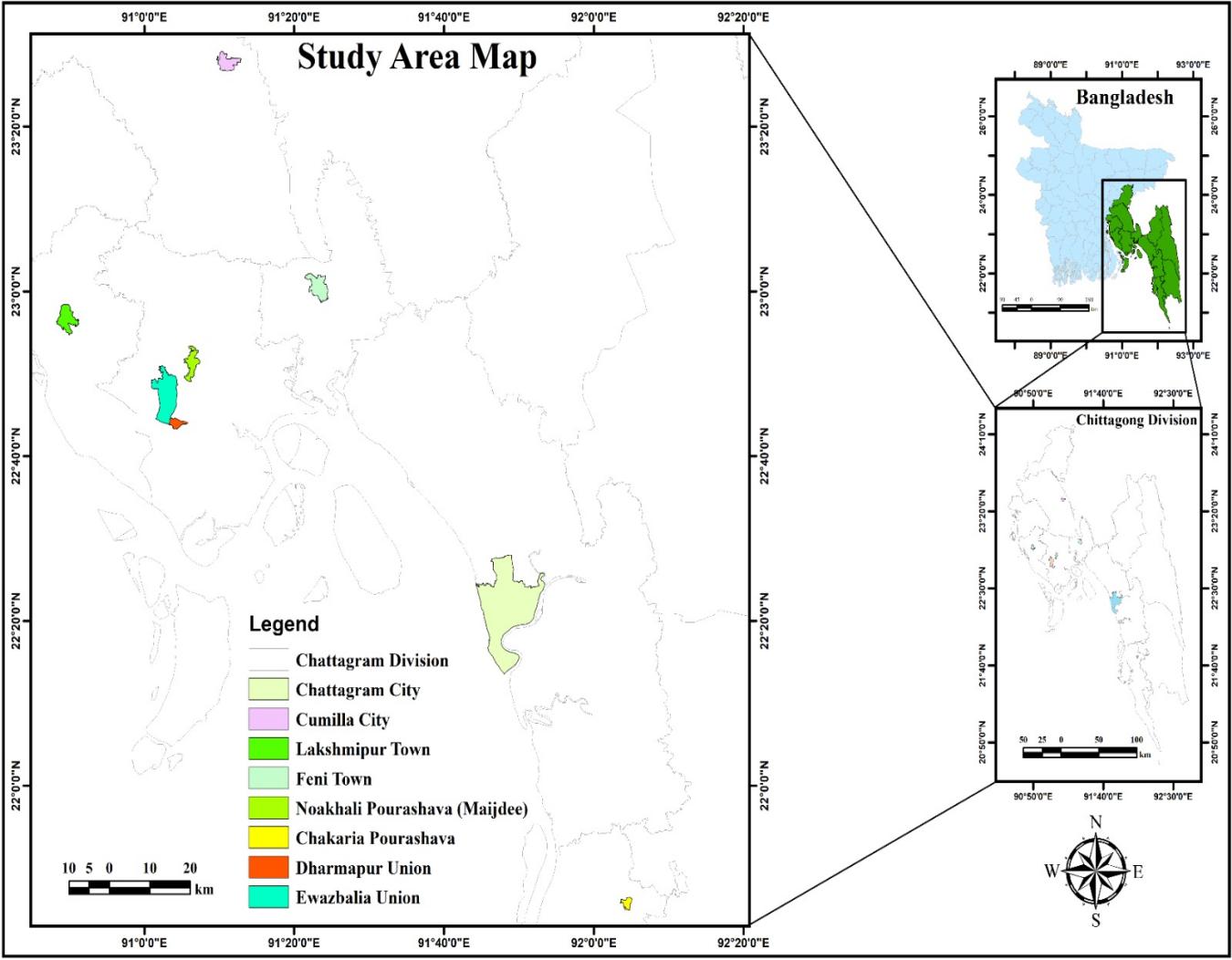
**

**Supplementary Figure-S1.** Area of study that represented the eight surveyed clusters (Prepared by ArcGIS version 10.8 and Google Earth Pro)

**Iodine Concentration Measurement Procedure (Organization, 2007):**

**Mechanism of reaction:**

**Iodometric titration includes two phases in its reaction:**

1. Free iodine is liberated from salt whenever sulfuric acid is added, which dissolves the iodate in the salt sample. Additional KI is added to assist in dissolving the free iodine, which is normally quite insoluble in pure water.
2. During the titration phase, sodium thiosulfate consumes free iodine. The thiosulfate utilized is proportionate to the amount of free iodine liberated by the salt. As an external indicator, starch is applied and reacts with free iodine to generate a blue colour. When thiosulfate is introduced at the end of titration, the loss of dark blue, or end-point, shows that all remaining free iodine has been absorbed by thiosulfate.

**Iodometric titration of iodate reaction steps:**

1. IO_3_  + 5l^-^ + 6H^+^ → 3I_2_  + 3H_2_O

(from Kl) (from salt) (from H2SO4)

1. 2Na_2_S_2_O_3_  + I_2_  → 2NaI + Na_2_S_4_O_6_

(Sodium thiosulfate) (Iodine) (Sodium Iodide) (Sodium tetrathionate)

**Reagent preparation:**

1. **0.005 M Sodium thiosulfate solution (Na_2_S_2_O_3_)**: 0.124 gm Na_2_S_2_0_3_5H20 dissolved in 100 ml water. I stored it in a cool, dark environment.
2. **Solution of 2 N Sulfuric acid (H_2_S0_4_):** Slowly add 6 mL of concentrated H**_2_**S0_4_ in 90 mL water, dissolved to 100 mL with water
3. **10% potassium iodide (KI) solution:** 10 gm of KI dissolved in 100 mL water and protected from direct sunlight.  Stored in a brown bottle in a cool-to-dark place
4. **1% solution of the starch indicator:** 1 gm of soluble starch was weighed, and warmed and stirred into 100 ml of double distilled water. The solution was heated to 90˚ Celsius for 5 minutes for five minutes. After completely cooled, the starch solution was filtrated through filter paper and delivered to a new volumetric container.

**Step of determining salt iodate and salt iodide content:**

- **Step 1:** A 10-gm sample of iodized salt was weighed using an electronic balancing scale. The measured salt was completely dissolved in 50 mL of distilled water.
- **Step 2:** Sulfuric acid is added after the measured salt and distilled water completely dissolve. Approximately 2 mL sulfuric acid and 5 mL potassium iodide were added to the salt solution. The solution turned yellow in the presence of iodine. To achieve the optimal reaction time, the reaction mixture is placed in a dark place for 10 minutes.
- **Step 3:** After 10 minutes in the dark, 2ml of 1% starch solution indicator is added to the yellow colored solution, and the color of the solution produces a dark blue-black colored complex with iodine.
- **Step 4:** The titration was titrated with sodium thiosulfate until the colour disappeared completely. The following formula is used to estimate the iodine content of salt based on the titrated volume (burette reading) of sodium thiosulfate.

**
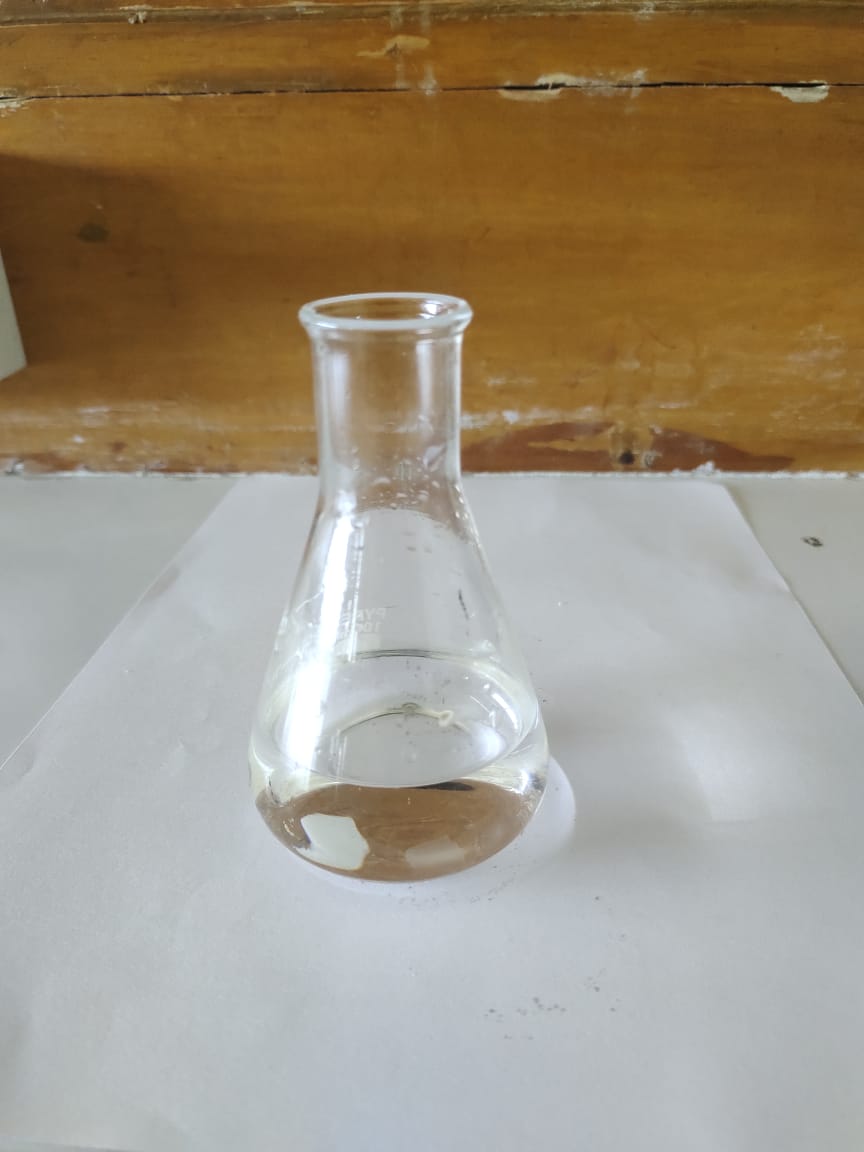

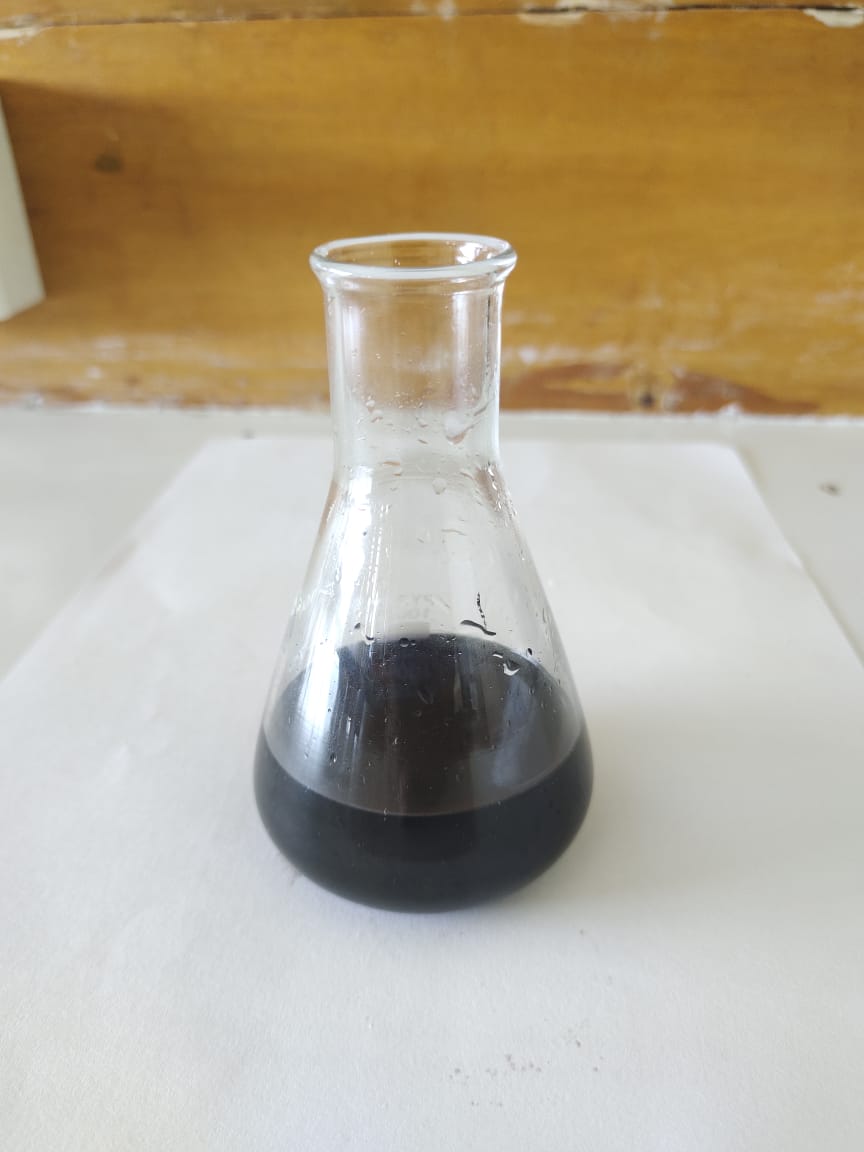

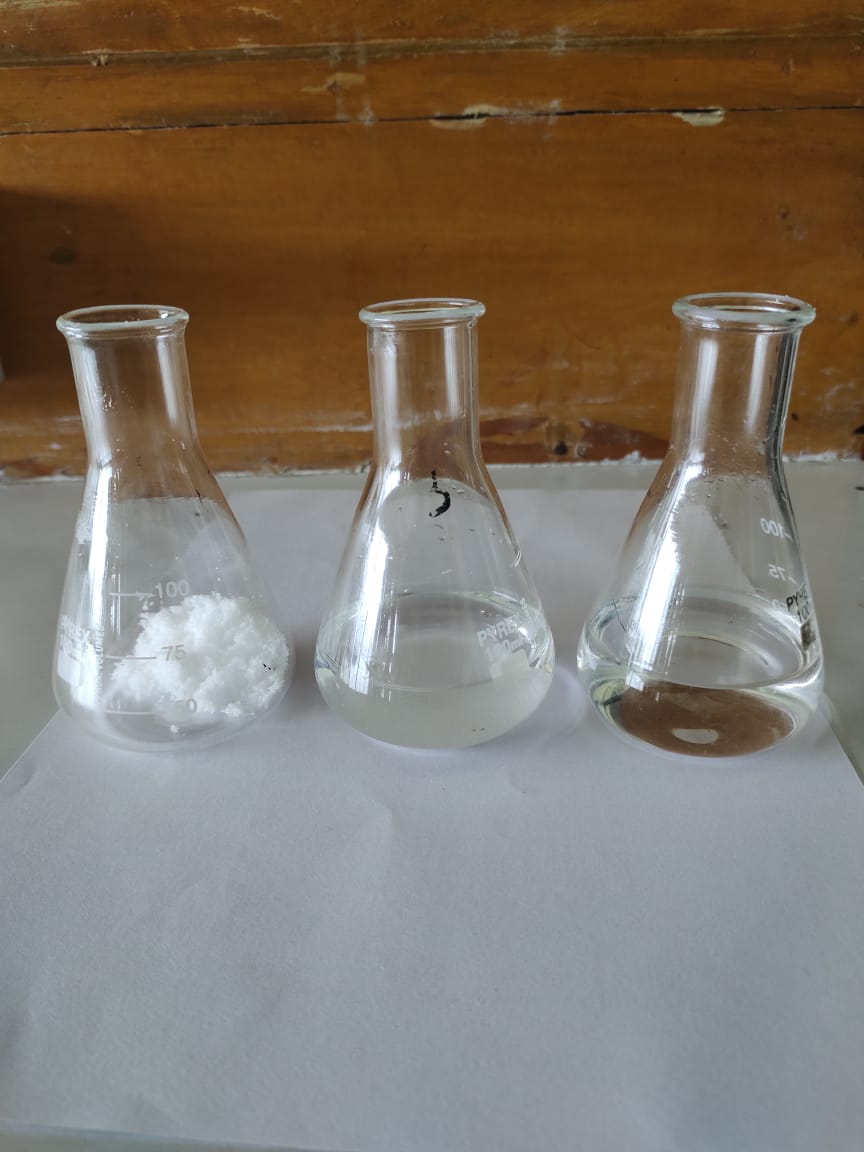
**

**Plate 1.** Iodometric titration method of the iodine in salt samples

**Calculation:** Mg/kg Calculation (ppm) Iodine = titration volume in ml x 21.15 x sodium thiosulfate normality x 1000 / salt sample weight in gm.

**References:**

ORGANIZATION, W. H. 2007. Assessment of iodine deficiency disorders and monitoring their elimination: a guide for programme managers.
